# Motivation in the metaverse: A dual-process approach to consumer choices in a virtual reality supermarket

**DOI:** 10.3389/fnins.2023.1062980

**Published:** 2023-02-16

**Authors:** Farzad Saffari, Shobhit Kakaria, Enrique Bigné, Luis E. Bruni, Sahar Zarei, Thomas Z. Ramsøy

**Affiliations:** ^1^Neurons Inc., Høje-Taastrup Municipality, Denmark; ^2^Augmented Cognition Lab, Aalborg University, Copenhagen, Denmark; ^3^Faculty of Economics, University of Valencia, Valencia, Spain

**Keywords:** electroencephalogram, virtual reality, frontal asymmetry, consumer neuroscience, decision making

## Abstract

**Introduction:**

Consumer decision-making processes involve a complex interrelation between perception, emotion, and cognition. Despite a vast and diverse literature, little effort has been invested in investigating the neural mechanism behind such processes.

**Methods:**

In the present work, our interest was to investigate whether asymmetrical activation of the frontal lobe of the brain could help to characterize consumer’s choices. To obtain stronger experimental control, we devised an experiment in a virtual reality retail store, while simultaneously recording participant brain responses using electroencephalogram (EEG). During the virtual store test, participants completed two tasks; first, to choose items from a predefined shopping list, a phase we termed as “planned purchase”. Second, subjects were instructed that they could also choose products that were not on the list, which we labeled as “unplanned purchase.” We assumed that the planned purchases were associated with a stronger cognitive engagement, and the second task was more reliant on immediate emotional responses.

**Results:**

By analyzing the EEG data based on frontal asymmetry measures, we find that frontal asymmetry in the gamma band reflected the distinction between planned and unplanned decisions, where unplanned purchases were accompanied by stronger asymmetry deflections (relative frontal left activity was higher). In addition, frontal asymmetry in the alpha, beta, and gamma ranges illustrate clear differences between choices and no-choices periods during the shopping tasks.

**Discussion:**

These results are discussed in light of the distinction between planned and unplanned purchase in consumer situations, how this is reflected in the relative cognitive and emotional brain responses, and more generally how this can influence research in the emerging area of virtual and augmented shopping.

## Introduction

Our everyday behaviors and choices result from a complex interplay between our perceptions, emotions, and cognition. Choices we make range from highly planned to direct and impulsive behaviors. This distinction is often seen in well-controlled studies in psychological and cognitive neuroscience. The distinction between decisions as ranging from cognitive to emotional responses has been investigated in various disciplines, i.e., neuroscience, philosophy, psychology, and commercial studies (Schall, 2001). Most notably, behavioral studies have long held a view of a split between an emotional and impulsive system, and a more cognitively driven deliberation system (Frankish, 2010). This has also been demonstrated in neuroimaging studies showing a distinction between two separate neural systems for immediate and delayed rewards (McClure et al., 2004).

However, less is known about the role of dual-process (an interplay between our emotion and cognition) decisions in real-life situations, especially in the ubiquitous digital reality in which consumer behaviors are embedded in our days. Numerous studies in neuroscience have been examining consumers’ shopping behavior (Martin and Potts, 2009; Ravaja et al., 2013; Gable et al., 2015; Ramsøy et al., 2018; Goto et al., 2019; Liao et al., 2019; Shih et al., 2019; Ozkara and Bagozzi, 2021). With the help of quantitative measurement in neuroscience such as electroencephalogram (EEG), these relatively vague concepts can be analyzed and furtherly explore consumer decisions. The most recent studies in neuromarketing have been focused on consumer’s purchase intention and decisions (purchase vs. no-purchase) (Shang et al., 2020; Wang et al., 2021), or predicting consumer’s purchases (Golnar-Nik et al., 2019; Bak et al., 2022). But fewer studies have gone beyond “buy” and “no buy” and the relative contribution of cognitive and emotional process on consumer motivation (Aditya and Sarno, 2018; Royo-Vela and Varga, 2022) and the distinction of consumers decisions have not been thoroughly investigated.

Human emotion plays a central role in our everyday decisions (Rutherford and Lindell, 2011; Harmon-Jones et al., 2012). Previous research has shown that our positive or negative perceptions could be explained by approach\avoidance behavior (Rutherford and Lindell, 2011; Harmon-Jones et al., 2012). When it comes to human shopping behavior, the reasoning behind our decision becomes more complex and difficult to explain (Ohme et al., 2010; Wixted, 2018; Karmarkar and Plassmann, 2019; Neal and Gable, 2019; Sänger, 2019).

Lateralized function of the brain has been the core of many neuroscientific studies on emotion (Petrantonakis and Hadjileontiadis, 2011; Gable et al., 2015), motivation (Coan et al., 1997; Poole and Gable, 2014), neurological and psychiatric disorders (Keune et al., 2015; Lin et al., 2021). Specifically, frontal asymmetry scores are one of the most promising features that have been extracted from EEG data to quantify our valence and approach-avoidance behavior (Coan et al., 1997; Davidson, 2004; Ravaja et al., 2013; Poole and Gable, 2014; Wixted, 2018).

Frontal asymmetry has been traditionally used by neuroscientists as an indicator of human emotions (Smith et al., 2017), which relies on the theory that the left prefrontal cortex is more activated for emotions with positive valence compared to those with negative valence, which induce relatively less left prefrontal activity. Moreover, the approach/avoidance behavior has been studied in this manner as a motivation index. Most of these studies have been focused on the alpha frequency band, which relates to the inhibitory function of the brain and a higher relative power in this band indicates less cortical activation (Smith et al., 2017). However, less attention has been paid to the other EEG frequency bands such as beta and gamma (Ravaja et al., 2013; Ramsøy et al., 2018; Spironelli et al., 2021).

The theta-alpha ratio (TAR) has been recently used as a cognitive load index which is sensitive to task difficulty and cognitive processing (Trammell et al., 2017; Cabañero et al., 2019; Wang et al., 2020). Here, we assumed that the TAR index would vary as participants switched their shopping behavior from decisions that were mainly emotion-based, and hence frontal asymmetry based, to a more cognitive state of planned behavior, which we expected to be related to stronger TAR scores.

Consumer behaviors have recently changed from being physically based in brick-and-mortar stores to moving online (Cheung et al., 2005), and finally now on the verge of moving fully into mixed reality (MR) (Shen et al., 2021). Recent research in Virtual Reality (VR) tried to combine VR and supermarket as a research tool (Peschel et al., 2022), or as a teaching application for Autistic patients (Thomsen and Adjorlu, 2021). Also, VR supermarkets have been used to investigate consumers’ behavior in choosing healthy food (Eichhorn et al., 2021; Melendrez-Ruiz et al., 2021), or with the help artificial intelligence, virtual supermarket has been studied as a “shopping at home” avenue (Shravani et al., 2021). However, to the best of our knowledge, the effect of virtual supermarkets on consumers’ subconscious response has not been thoroughly investigated *via* psychophysiological tools such as EEG. Thus, there is a need for a better understanding of consumer behaviors and relative underlying neural responses in general, as well as in the new digital interfaces that consumers are currently moving into.

Recently, different effects of VR on our brain responses have been measured through EEG (D’Errico et al., 2020; Dini et al., 2022). Due to VR engagement and the semi-real environment that these technologies provide, they could facilitate examining neural responses in a more realistic way. In Schaefer et al. (2016) a VR shopping task has been conducted to investigate the effect of price expectation violation on the P300 component of EEG and in Rosenlacher et al. (2018) the effects of a VR store on human shopping behavior have been studied. Besides consumer neuroscience, VR and MR have been used as a treatment tool (Tran et al., 2022), or as an educational avenue (Makransky et al., 2019) in neuroscientific studies. Specifically, as there is now a surge in interest for implementing MR worlds as a solution for everyday situations (Bazzani et al., 2020)—increasingly referred to as the “metaverse”—our knowledge about attentional, emotional, and cognitive responses is woefully lacking. While we can, *a priori*, assume that the responses we see inside MR are the same as those that we see outside MR, few studies have been conducted to test this assumption. Hence, although numerous neuroscientific studies tried to investigate the neural mechanism behind consumers’ decision-making process, the validity of the finding of those studies have not been tested in a real-life scenario.

In this study, we designed an experiment in a VR supermarket, with minimal experimental control and high ecological validity, to investigate whether there is a difference in consumers’ relative involvement of cognitive and emotional processes in planned vs. unplanned decisions, respectively. Participants went through two experimental phases while being in the VR store. First, they were asked to buy products following a predefined shopping list. Second, they were instructed to also buy whatever they wanted from the store.

We posed two main hypotheses. First, following prior research on frontal asymmetry and choice, we expected stronger frontal asymmetry responses when consumers made choices, compared to phases of product inspection with no choice, regardless of whether the choice was planned or unplanned. More specifically, frontal asymmetry, as a representation of participants’ approach behavior, is assumed to be higher in choice trials compared to the no-choice trials, i.e., periods of the experiment in which participants are navigating or searching for products.

Second, we assumed that planned purchases would rely more on cognitive brain responses implying higher cognitive load, while spontaneous, unplanned purchases would be more driven by emotional brain responses. Therefore, we hypothesized that unplanned purchases would be related to a stronger frontal asymmetry score during product choice, compared to planned purchases. Conversely, we hypothesized that planned purchases would be associated with a relatively lower frontal asymmetry and a higher degree of cognitive load.

## Materials and methods

A total of 30 (14 female, 16 male) right-handed subjects (age range 23–44, mean = 31.8, std = 6.6) were recruited in the experiment using Neurons Inc^1^ online recruitment procedures. Since we aimed to compare the EEG responses in two conditions, we only included the data from the subjects who fulfilled both tasks in the experiment which reduced the number of subjects to 27 (age range 23–44, mean = 31.6, std = 6.9). From these participants that we consider the data for the study, eight of them have previous experience with VR and using the controllers and two of them have participated in a study with virtual supermarket previously. All participants read and signed the consent form, and they were informed about the experimental procedure prior to the data collection. This study was performed in accordance with the Declaration of Helsinki, the rules and laws of the Danish Data Protection Agency, the European Union law of the General Data Protection Regulation, as well as the ethical regulations imposed by the Neuromarketing Science and Business Association, Article 64. Each person’s biometric data, survey responses, and other types of data were anonymized and only contained the log number as the unique identifier. No personally relevant data could be extracted from the log number.

### Experimental design

**FIGURE 1:**
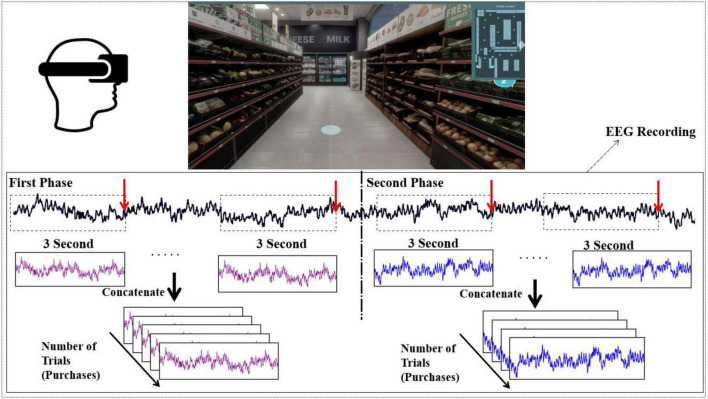
The environment for virtual supermarket and EEG data collection procedure is presented. The red arrows show the time points in which they choose a product. We consider the 3 s data before that event as a trial. Afterward the trials for each condition have been concatenated for further analysis.

A supermarket environment was designed in Unreal Engine V4.1^2^ and implemented in a VR HTC Vive 5.^3^ The VR supermarket was comparable to the supermarkets in Denmark in terms of appearance. Participants had two controllers (which appeared as hands in the VR) that they can use to choose the items and teleport through the VR. They could also walk (for one or two steps in the physical space), but we asked them to limit their movement in order to keep them in VR environments. All of the items in the supermarket were accessible and participants could choose whatever they see in the store through free navigation. We have allocated a budget of 250 Danish Kroner (DKK) (∼$35) for each participant to spend on these two shopping tasks. Therefore, participants were free to distribute their budget between these two shopping tasks and prevent the neurological effect of product price suggested by Tang and Song (2019) in online context. We provided a list of products in the VR for the participants, which contained six items (Broccoli, Milk, Cheese, Soda, Cereal, and Chocolate). The range of the cost of these items was between 80 and 120 DKK. Participants need to purchase all of these items, which we call as planned purchase condition. With the remaining amount of budget, they could buy whatever they wanted in the store (Unplanned Purchase Condition). They could leave the environment as soon as they had bought the items they wanted. The number of planned purchases (items from the list) was fixed, and the number of unplanned purchases could be different among the subjects, as they were free to choose any item they wanted if it was within their budget. In Figure 1, the experimental procedure of the experiment is illustrated. In the upper side of the figure, the environment for the virtual supermarket is presented.

The EEG data were recorded during the whole shopping task. After mounting the VR and EEG cap, we instructed the participant on how to use the VR so they could start the experiment and navigate through the store. The list of the products that they needed to purchase was available for them by hitting a button on the controller. In the first phase, they were required to buy all the products from the list. Afterward, we informed them about the remaining budget. Then, they could start the second phase of the experiment. On average, participants spent 238.87 ± 85.57 s on the planned purchase condition, and 228.00 ± 107.20 s on the unplanned purchase condition. The time-points of each purchase (i.e., when the participants choose an item) have been recorded during the experiment.

### EEG recording

Electroencephalogram data were collected using a monopolar 32 channels (gel-based) EEG device (Brain Products, ActiCap) with 500 Hz sampling rate. The EEG sensor locations with the standard 10–20 system were positioned at Fp1, Fp2, F8, F4, Fz, F3, F7, FT9, FT10, FC5, FC1, FC2, FC6, T7, T8, C3, Cz, C4, CP5, CP1, CP2, CP6, TP9, TP10, P7, P3, Pz, P4, P8, O1, Oz, O2. The ground and reference electrodes were located at Afz and FCz, respectively, for online processing. The data were transmitted wirelessly from the amplifier (LiveAmp32) to a PC using a USB module. The impedance of the electrodes was monitored prior to the data collection, and it was maintained below 15 KΩ.

### EEG analysis

For the EEG data analysis, we used the MNE tool (Gramfort, 2013). First, the data were filtered using a FIR bandpass filter with a hamming window for the 0.1 and 100 Hz frequency bands. A notch filter at 50 Hz was also applied to remove the power line noises. Independent Component Analysis (Jung et al., 1998) was applied to clean the data and manually remove the “bad” components by visual inspection. Afterward, common average referencing and baseline correction methods were used for pre-processing procedures.

Based on a recent study (Ramsøy et al., 2018), we considered 3 s epochs of the data before each participant chose an item (in both conditions) for our analysis. The rest of the data, which was not included in choosing the products was also epoched with 3 s length for the consistency of the analysis. The power spectrum of the signal was calculated using a welch method (with 256 numbers of FFT points equivalent to 512 milliseconds) for each frequency band (theta [4–8], alpha [8–13], beta [13–25], gamma [30–40]). For each epoch, the power spectrum was calculated by averaging over frequency bins and then averaged over all epochs to represent the power spectrum of that condition for each subject.

### EEG feature extraction

#### Frontal asymmetry

The frontal asymmetry score is a well-established EEG feature to indicate the lateralization effect of emotional processing in the brain (Lee et al., 2020; Lin et al., 2021; Yang et al., 2021). Frontal asymmetry is calculated by subtracting the log-transformed power values of frontal channel F7 from frontal channel F8 and divided by the sum of the power of both electrodes, as shown in Equation 1. Frontal asymmetry was measured for alpha, beta, and gamma frequency bands. It should be noted that the alpha scores are not multiplied by −1 to indicate the “de-activation” of the alpha band.


(1) $$\frac{L\,o\,g\,\left(p\,o\,w\,e\,r\,\left(F\,7\right)\right)-L\,o\,g\,\left(p\,o\,w\,e\,r\,\left(F\,8\right)\right)}{L\,o\,g\,\left(p\,o\,w\,e\,r\,\left(F\,7\right)\right)+L\,o\,g\,\left(p\,o\,w\,e\,r\,\left(F\,8\right)\right)}$$

#### Theta-alpha ratio

TAR indexes the cognitive load value by using cross-frequency activity in the frontal and parietal lobes. As shown in Equation 2, power values in the theta frequency band from F7 and F8 channels were normalized to power values in the alpha frequency band at the parietal lobe (P3 and P4) (Wang et al., 2020).


(2) $$\frac{Power\left(F7\left(theta\right)\right)+Power(F8\left(theta\right)}{Power\left(P3\left(alpha\right)\right)+Power(P4\left(alpha\right)}$$

### Statistical method

**FIGURE 2:**
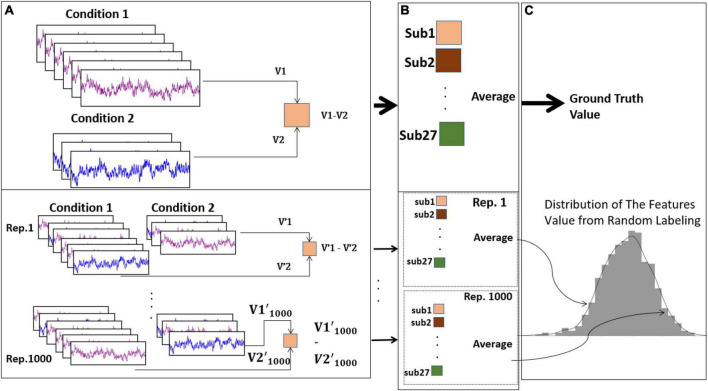
The implemented statistical method for considering the imbalanced trials. In section **(A)**, the calculation of feature values has been shown. In the upper section, features from the actual conditions (V1, V2) have been calculated and then subtracted for each subject. In the lower section of **(A)** the same procedure is happening but with random shuffling of the labels across trials for 1,000 iterations. In section **(B)** we averaged the outcome of section **(A)** over subjects which the upper section shows for the actual condition and the lower section is for each iteration. In section **(C)** the ground truth value from the actual conditions and null distribution from the random shuffling is illustrated in the upper and lower side respectively.

As described in the experimental design section, we asked each participant to purchase six items from a predefined list, and then they could purchase whatever they wanted with the remaining budget. Accordingly, this led to an imbalance of trials, both within each subject (between each condition) and between subjects. To solve this imbalance, we applied a permutation test, which previously has been applied to resolve the imbalance of trials issue in an ERP study (Files et al., 2016). As shown in Figure 2, for each participant, we first calculated the desired feature (asymmetry score in different frequency bands) from the trials of each condition. We subtracted those values of one condition from the other to calculate the difference between the features. By repeating this procedure for all subjects, we computed the average of these values for all the participants to build up the “ground truth” values for that comparison.

Next, for generating data-driven null distribution, we randomly shuffled the trial’s labels between conditions for each subject, and then repeated the same procedure that we did to calculate the ground truth. By performing this procedure 1,000 times, we could have a null distribution to compare the ground truth value in the two conditions. Ultimately, if the ground truth is far enough (based on the significance level which here is 0.05 before Bonferroni’s correction) from the mean of this distribution, we could state that our findings are not due to randomness. Due to multiple comparisons, the significance level is 0.05/3 = 0.01.

We applied this statistical method to compare the asymmetry scores in alpha, beta, and gamma frequency bands, in the two given conditions: purchase vs. non-purchase, and planned purchase vs. unplanned purchase.

## Results

### Frontal asymmetry

**FIGURE 3:**
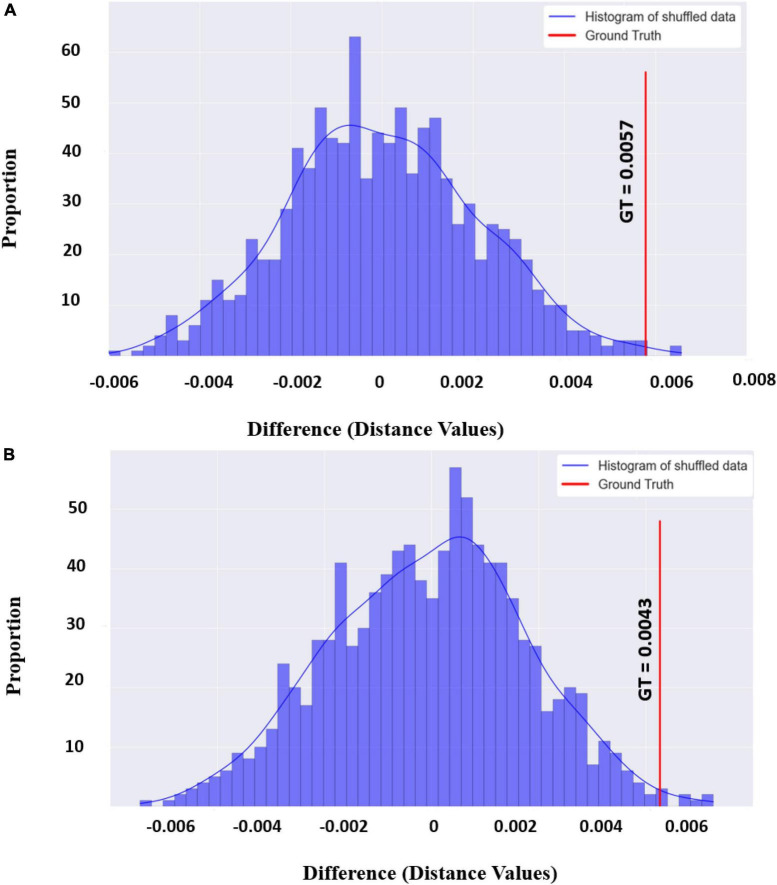
**(A)** The distribution and ground truth values of the beta asymmetry score differences in the two conditions (purchase vs. Non-purchase). The distribution derives from calculating the difference of the beta asymmetry score between two conditions for 1,000 times and shuffling the labels among trials. The blue vertical line is our ground truth which indicates the same difference but for the actual conditions. **(B)** Same as the above plot but for planned and unplanned conditions in gamma frequency band.

Looking at the distinction between purchase and non-purchase conditions, we find a significant difference in alpha, beta, and gamma asymmetry scores (F7, and F8 channels) between the two conditions. The results of the beta asymmetry scores for these two conditions are presented in Figure 3A as an illustrative example to show the significance level (*P*-value < 0.01). For the rest of the frequency bands, the significance plots were similar to Figure 3, therefore, we are not presenting the plots here to avoid repetition. As it is shown in Figure 3, the ground truth of asymmetry scores was far from the mean of null distribution which makes the ground truth value statistically significant.

**FIGURE 4:**
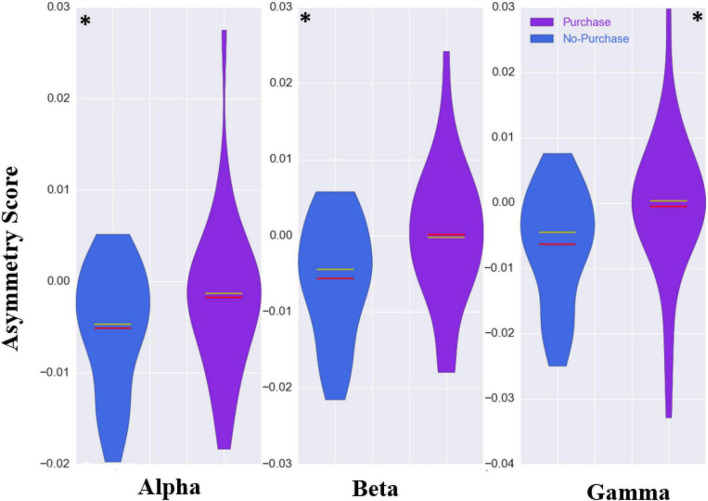
The violin plots of asymmetry scores of three frequency bands for all subjects. The purchase and non-purchase conditions are shown with blue and purple colors, respectively. The red and yellow line in the middle illustrating the mean and median values, respectively. The difference between the asymmetry scores were statistically significant (*p-value < 0.001) in all tree frequency bands.

In the alpha frequency band, the asymmetry mean scores for purchase and non-purchase conditions over all subjects were negative and the score for the purchase phase (−0.001 ± 0.008) was higher (*p*-value < 0.001, 1000 permutations) than the non-purchase phase (−0.005 ± 0.006). For beta and gamma frequency bands, the mean asymmetry scores were positive for the purchase phase (0.0001 ± 0.009 and 0.0008 ± 0.011) and negative for the non-purchase phase (−0.005 ± 0.007 and −0.006 ± 0.008) with *p*-value < 0.001 and 1,000 permutations. The violin plots of these results are presented in Figure 4.

**FIGURE 5:**
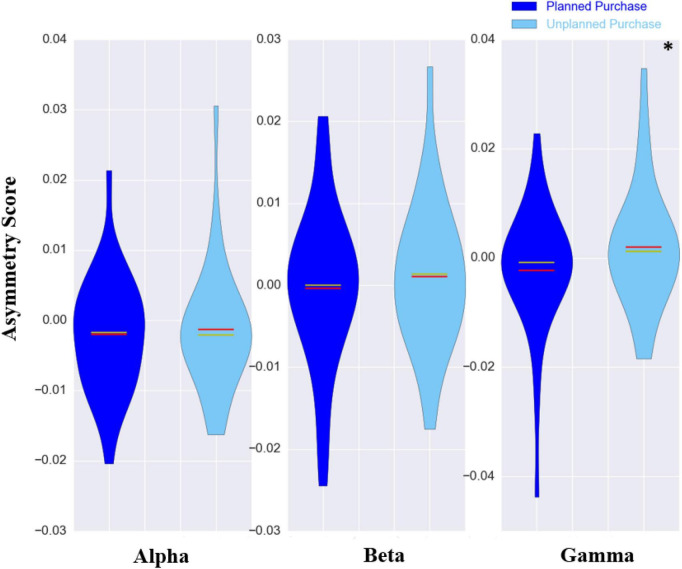
The violin plots of asymmetry scores of three frequency bands for all subjects. The planned purchase and unplanned purchase conditions are shown with dark-blue and light-blue colors, respectively. The red and yellow line in the middle illustrating the mean and median values, respectively. The difference between the asymmetry scores was statistically significant (*p-value < 0.001) in gamma frequency band.

For the comparison between planned and unplanned purchase, there was a trend toward a significant difference in the range of alpha (*P*-value = 0.08) and beta (*P*-value = 0.04) asymmetry scores before correction, between these two conditions. However, in the gamma band, the difference between mean asymmetry scores of the unplanned purchase (0.001 ± 0.011) and planned purchase (−0.002 ± 0.012) was statistically significant (*P*-value < 0.001, 1000 permutations). The results of the statistical method for the gamma frequency band are presented in Figure 3B, and the violin plots of asymmetry scores for different frequency bands are presented in Figure 5.

**FIGURE 6:**
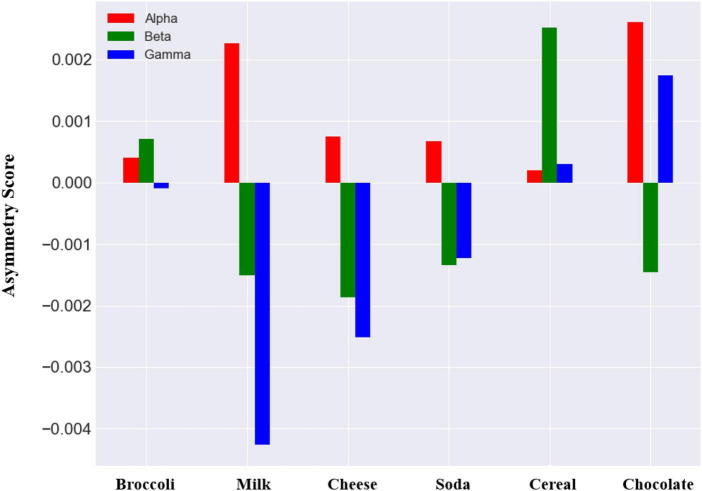
Asymmetry scores in alpha, beta, and gamma frequency bands for each item in planned purchase. Asymmetry scores in alpha have been multiply by negative 1 to represent a same behavior as other frequency bands.

The asymmetry scores in alpha, beta, and gamma frequency bands for each of the products in planned purchase are presented in Figure 6 (averaged for all subjects). For each of the six products, the alpha asymmetry scores are positive (considering the multiplication by −1) and chocolate has the highest alpha asymmetry. For beta and gamma asymmetry, the scores are both negative and positive. Cereal and chocolate have the highest beta and gamma asymmetry scores respectively.

**FIGURE 7:**
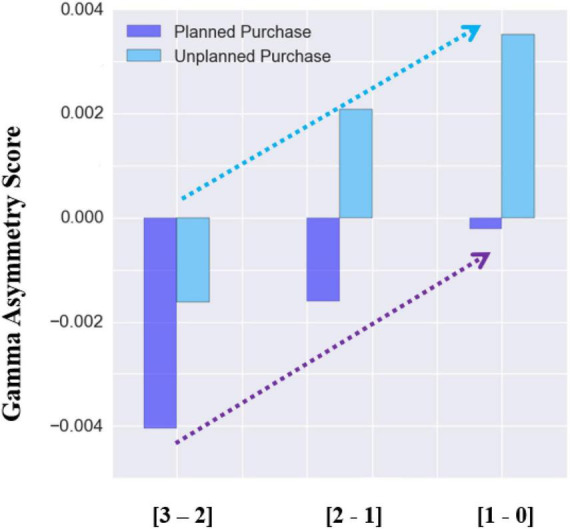
Gamma asymmetry score (averaged for all subject over all purchased items) for the three 1-s epochs before choosing a product. The horizontal axis shows the three time-intervals (within the 3 s window) before choosing a product.

In addition, we have compared the asymmetry score in the gamma frequency band of each second (from the 3-s time window) before choosing an item. As it is shown in Figure 7, for both planned and unplanned purchases, as participants become closer to choosing an item, the gamma asymmetry score increases. For unplanned purchase, the asymmetry score starts from negative and become positive when there is a 2 s before choosing an item. However, for the planned purchases, even though the asymmetry score increased as we get closer to choosing a product, the asymmetry score remains negative for each of the three phases before choosing an item.

### Theta alpha ratio

**FIGURE 8:**
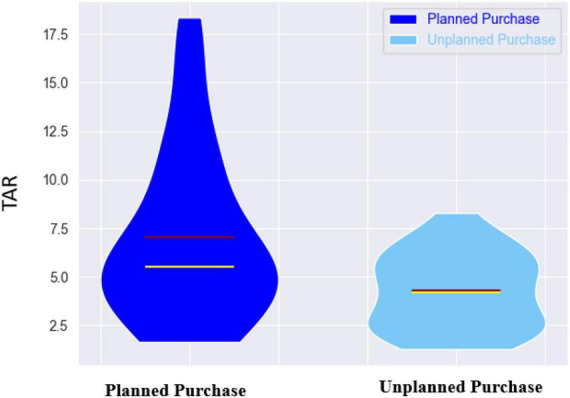
The violin plots of TAR index across all subjects. The planned purchase and unplanned purchase conditions are shown with dark-blue and light-blue colors, respectively. The red and yellow lines in the middle illustrate the mean and median values, respectively.

Comparing TAR values of planned and unplanned purchase, we found a significant difference between the given conditions with *p*-value < 0.0001. As represented in Figure 8, the averaged TAR index over all subjects was 7.04 ± 4.35 for planned condition and 4.28 ± 1.92 for unplanned condition.

## Discussion

In this study, we sought to test whether consumer choices in a VR supermarket can modulate brain frontal asymmetry in alpha, beta, and gamma frequency bands. In addition, we were interested in testing whether planned and unplanned choices would be related to similar changes in emotional and cognitive responses such as frontal asymmetry and TAR, respectively.

In doing so, we found that regardless of the types of participants’ purchases, there was a significant difference in alpha, beta, and gamma asymmetry scores comparing trials that involved choosing a product, compared to other phases of store task completion, such as navigating the environment. We also showed that different types of consumer choices (planned and unplanned), were related to a modulation of the gamma asymmetry scores but not the alpha or beta frontal asymmetries.

Our findings for comparison of “choice vs. no-choice” were partially in line with the previous studies regarding frontal asymmetry and approach behavior. Previous research has shown that since alpha oscillation is more correlated with inhibitory activities, relatively higher right frontal activity is related to the approach behavior in this frequency band (Harmon-Jones and Allen, 1998; Pizzagalli et al., 2005; Ravaja et al., 2013; Smith et al., 2017). However, our results showed a contrast in alpha frontal asymmetry, in which there was a relatively higher right frontal activity in the no-choice phase. For the beta and gamma frequency bands, there was a relatively higher frontal asymmetry during the “choice” conditions, which was in line with previous research that relates higher left frontal beta activity compared to right frontal activity to consumers’ purchase decisions (Aftanas et al., 2006; Ramsøy et al., 2018; Zhao et al., 2018; Alarcão and Fonseca, 2019; Lin et al., 2021; Yuan et al., 2022).

Our second hypothesis was concerned with the comparison between planned and unplanned choices. Here, our results confirmed our hypothesis as there was a significantly higher left than right frontal activity in the gamma frequency band during unplanned conditions, relative to planned choices. As some recent studies suggested (Ramsøy et al., 2018; Tarrant and Cope, 2018), we found a greater gamma asymmetry score for unplanned choices compared to planned choices. These findings provide new insights into the role of gamma frequency band activity in decision making particularly in consumer neuroscience.

Moreover, when looking at the cognitive index of the TAR score for the planned and unplanned conditions, our results show that planned purchases were associated with relatively higher levels of the cognitive load than unplanned purchases. This is in line with our assumption and prior findings and suggests that planned behavior is reflected as a theta-alpha activity ratio even in a virtual shopping environment. As opposed to most of the research in the field of consumer neuroscience, which mainly focus on consumers’ “purchase” and “no purchase” (Rosenlacher et al., 2018; Golnar-Nik et al., 2019; Goto et al., 2019; Eichhorn et al., 2021; Melendrez-Ruiz et al., 2021; Horr et al., 2022) we have extended this view by looking deeper to the relative contribution of cognitive and emotional response and the dual-process of consumer’s decision-making process. Additionally, one of the important contributions of this study is that, compared to highly controlled lab experiments, it adds a higher ecological validity to the research by using an immersive VR supermarket. Most previous studies that have investigated emotions and frontal asymmetry have been customarily conducted in lab settings. However, with the current study we can expand the interpretation more widely into our daily decisions. Also, little is known about the effects of MR systems on human emotional and cognitive responses. The present research shows that those findings can be partially reproduced in a VR-based experiment which is a closer simulation of real-life conditions.

As for the limitations of this study that also prompt future research, it should be noted that these findings should be tested in other VR settings to eliminate the sensitivity of the results to a specific environment. Additionally, other studies should seek to do a direct comparison of virtual and real store environments to ensure that there is indeed a high degree of reliability of the identified emotional and cognitive scores across environments. In addition, although the feedback we got from the participants was overally positive, there were some limitations to the environment (e.g., they could not carry the products to the cashier, or they could not find some types of products in the supermarket) which should be improved and lead us to novel hypotheses such as the role of product type on frontal asymmetry.

Another limitation of this is study is that we did not consider personal preferences (which lead to a higher motivation) of the participants for each product. Collecting the data on personal preferences for each product that participants have bought within both planned and unplanned purchases, a comprehensive comparison of participants’ motivation resulted from EEG for each item and its relation to their behavioral response could be conducted.

Another limitation of this study is the imbalance of trials and fewer number of trials for one condition. This limitation was mainly because of our attempt to keep a high degree of ecological validity and thereby providing a shopping experience as realistic as possible for a daily shopping routine. Also, we sought to limit the experiment duration, as we had to consider elements of fatigue which is often caused by staying in a VR environment for a relatively long time. In our analysis, we fill this “gap” with the aforementioned statistical methods, to overcome the imbalance trials comparison. To further abate this, another solution could be to increase the number of participants and make a between-subject study. By doing so, there would be more trials for each condition, and the outcome of the statistical analysis would be less affected by imbalance trials. As a result, the participants would be less likely to experience fatigue during the VR experiments.

## Conclusion

In conclusion, we investigated the role of frontal asymmetry and TAR in explaining consumer choices in a virtual store. Although most previous studies have focused on frontal asymmetry in the alpha frequency band, we showed that information in the gamma band can explain consumers’ behavior more precisely. By conducting an experiment in a VR supermarket and two shopping tasks (planned and unplanned purchases) first we compared trials related to consumer choices and trials related to their navigation and searching products. We found a clear difference in alpha, beta, and gamma frontal asymmetry. In addition, we compared trials within the consumer choices (planned and unplanned choices), and results showed that gamma information in frontal asymmetry and TAR could discriminate those choices, which neither alpha nor beta frequency information would be apt to explain the difference.

The presented study tested previous findings related to frontal asymmetry in the consumer decision-making process. In addition, we confirmed previous finding in a semi-realistic VR environment, which makes the current study fruitful for consumer neuroscience and the VR research field. These findings and further research on the dual-process nature of consumer choices in MR systems will become increasingly important as emerging technological paradigms such as the Metaverse become distributed at the level that non-immersive social media have reached today.

## Data availability statement

The raw data supporting the conclusions of this article will be made available by the authors, without undue reservation.

## Ethics statement

The studies involving human participants were reviewed and approved by the Technical Faculty of IT and Design, Aalborg University. The patients/participants provided their written informed consent to participate in this study.

## Author contributions

FS was responsible for designing the experiment, collecting the data, pre-processing and analyzing the data, interpreting the result, writing the manuscript, and preparing the figures. SK was being responsible for designing the experiment and collecting the data. EB worked on the study design and contributed to the manuscript. LB and SZ contributed to the manuscript. TR was responsible for study design and interpreting the results contributed to the manuscript. All authors contributed to the article and approved the submitted version.

## Footnotes

1
https://www.neuronsinc.com

2
https://www.unrealengine.com

3
https://www.vive.com
